# A High Throughput Screen for RGS Proteins Using Steady State Monitoring of Free Phosphate Formation

**DOI:** 10.1371/journal.pone.0062247

**Published:** 2013-04-23

**Authors:** C. Aaron Monroy, Duncan I. Mackie, David L. Roman

**Affiliations:** 1 Division of Medicinal and Natural Products Chemistry, Department of Pharmaceutical Sciences and Experimental Therapeutics, University of Iowa College of Pharmacy, Iowa City, Iowa, United States of America; 2 Cancer Signaling and Experimental Therapeutics Program, Holden Comprehensive Cancer Center, University of Iowa Hospitals and Clinics, Iowa City, Iowa, United States of America; BioScience Project, United States of America

## Abstract

G-protein coupled receptors are a diverse group that are the target of over 50% of marketed drugs. Activation of these receptors results in the exchange of bound GDP for GTP in the Gα subunit of the heterotrimeric G-protein. The Gα subunit dissociates from the β/γ subunits and both proceed to affect downstream signaling targets. The signal terminates by the hydrolysis of GTP to GDP and is temporally regulated by Regulators of G-protein Signaling (RGS) proteins that act as GTPase Activating Proteins (GAPs). This makes RGS proteins potentially desirable targets for “tuning” the effects of current therapies as well as developing novel pharmacotherapies. Current methods for evaluating RGS activity depend on laborious and/or expensive techniques. In this study we developed a simple and inexpensive assay for the steady state analysis of RGS protein GAP activity, using RGS4, RGS8 and RGS17 as models. Additionally, we report the use of RGS4 as a model for high throughput assay development. After initial setup, this assay can be conducted in a highly parallel fashion with a read time of less than 8 minutes for a 1536-well plate. The assay exhibited a robust Z-factor of 0.6 in a 1536-well plate. We conducted a pilot screen for inhibitors using a small, 2320 compound library. From this screen, 13 compounds were identified as compounds for further analysis. The successful development of this assay for high-throughput screening provides a low cost, high speed, simple method for assessing RGS protein activity.

## Introduction

G-protein coupled receptors (GPCRs) are a diverse group of seven transmembrane-spanning receptors that represent targets for over 50% of drugs available on the market [1]. These receptors signal through the activation of a heterotrimeric G protein complex, consisting of G α, β, and γ subunits. Upon activation of the receptor, bound guanosine-diphosphate (GDP) is exchanged for guanosine-triposphate (GTP) in the Gα subunit. This causes a dissociation of the Gα subunit from both the receptor and Gβγ subunit complex, and both the Gα subunit and the Gβγ complex proceed to activate their respective signaling pathways. The signal is terminated by the hydrolysis of GTP to GDP in the Gα subunit [2]. The intrinsic, relatively slow rate of hydrolysis of the Gα subunit is temporally modulated by another superfamily of proteins, regulators of G-protein signaling (RGS) proteins, that increases the GTPase rate of a variety of Gα subunits, thus acting as GTPase activating proteins (GAPs) [3].

Due to their important role in regulating GPCR signaling, RGS proteins represent intriguing targets for drug development. In developing high-throughput screening (HTS) assays for RGS targets, methods have emerged for the targeting of the RGS-Gα protein-protein interaction, such as flow cytometry, Alpha Screen, fluorescence polarization, and time-resolved fluorescence resonance energy transfer [4]–[7]. These methods have been successfully used to detect the disruption of the protein-protein interaction and not the GAP functionality of the RGS proteins. Historically, the predominant method for determination of RGS protein activity is the use of ^32^P labeled GTP in single turnover or steady-state assays [8], [9]. While these ^32^P assays provides a measure of RGS activity on GTPase activity, they are technically challenging, even in low throughput benchtop experiments which involve the use of radioactivity and required careful timing for reproducible results [10].

**Figure 1:**
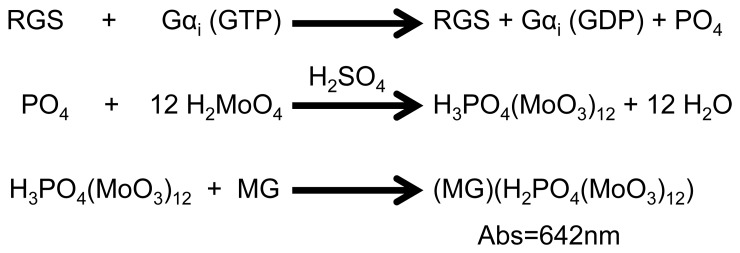
Scheme of Malachite Green Assay. RGS protein interacts with Gα_i_ and induces the hydrolysis of GTP to GDP, releasing free phosphate. In the presence of acid, molybdate releases water and complexes with the free phosphate. Lastly, the phosphomolybdate complex associates with the malachite green to produce a strong absorbance peak at 642 nm.

The limitations of these approaches have driven our group, and others, to develop simple, non-radioactive assays to measure RGS protein GAP function. Early work focused on the development of entire receptor/protein complexes contained within phospholipid vesicles [11]. This method is laborious and does not extend well into development of HTS assays. In order to develop a viable HTS assay for measuring GAP function, two hurdles must be overcome. First, the catalytic activity of the Gα subunit must be slowed to allow for a larger time window. Second, the rate-limiting step of Gα subunit turnover must be shifted from GDP dissociation to GTP hydrolysis. Analysis of the Gα subunit resulted in the previous reports describing a point mutation at the catalytically critical arginine residue (R178C in Gα_i1_) that results in a marked reduction in the intrinsic GTPase activity of the Gα subunit while maintaining sensitivity to the GAP activity of RGS proteins [8], [12], [13]. Another point mutation, A326S in Gα_i1_, allows for a ∼25 fold increase in k_off(GDP)_ while maintaining normal GTPase activity [14], [15]. These two point mutations have been used in the development of another HTS assay, the Transcreener assay (BellBrook Labs; Fitchburg, WI) to detect GDP generation [6]. The Transcreener assay relies on the usage of antibodies for the detection of generated GDP by fluorescence polarization. While this assay is well validated and commercially available, the use of antibodies in HTS assays can become prohibitively expensive. Therefore, we approached a very simple method previously used for detecting ATPase activity - the detection of free phosphate generation by a malachite green reagent [16]. As demonstrated in (Figure 1), free phosphate complexes with molybdate to form a phosphomolybdate complex called phosphomolybdic acid [17]. This phosphomolybdate complex then interacts with malachite green to develop an intense absorbance peak at 630 nm [18]. A single step addition method of this assay is accomplished by using low pH to improve the solubility of malachite green [19].

In this study, we developed a malachite-based assay to measure GAP activity of a variety of RGS proteins. RGS4 was selected as the pilot RGS for this assay due to the results of recent RGS4 HTS campaigns and the availability of a small collection of control compounds [4], [6], [7], [20]–[22]. While the majority of known RGS4 inhibitors act as irreversible cysteine modifiers (particularly at CYS148), our group, and others, seek the development of non-covalent RGS inhibitors [23]. The development of reversible inhibitors of RGS4 is of particular interest to the study of Parkinson’s disease (PD). Recent research has shown that RGS4 induction is an integral component of the progression of motor symptoms in mouse models of PD [24]. For this reason, in the development of the assay we include a counter screen against the cysteine null mutant of RGS4 (designated Δ7) to eliminate compounds that modify free thiols as their mode of inhibition [21]. This malachite green based assay allowed us to perform steady state analysis of RGS4, RGS8 and RGS17 activity readily in a plate based assay, acquiring data in as little as 40 min, with stability out to 2 h. After development, the absorbance remains stable for at least 30 min after, allowing for multiple reads of the same plate, such as scanning the fainter peak at 405 nm in order to evaluate compounds with strong absorbance at the principle peak of 630 nm [19]. Another benefit of this assay is the negligible cost of performing this assay, at approximately $0.005/well.

## Methods

### Expression and Purification of Recombinant Protein

Tobacco etch virus (TEV) protease was expressed and purified as a His-tagged protein in E. coli, BL21-pRIL (Stratagene; La Jolla, CA), in the pRK793 vector as previously described by the Waugh lab [25].

Rat RGS4, sharing 97% sequence identity with human RGS4, and the cysteine to alanine mutant were expressed as fusion proteins of maltose binding protein (MBP), a 10× His tag, and a TEV protease recognition site fused to the N-terminus of an RGS4 construct containing amino acids 51–205, in the vector pMALC2H10T in BL21-DE3 E. coli (Stratagene; Santa Clara, CA) [26]. The single cysteine-null Δ51-RGS4 construct was generated by site-directed mutagenesis as described previously [23]. Expression and purification were performed as described previously [23]. Purified protein was incubated with TEV protease at a molar ratio of 10∶1(fusion protein:TEV protease) overnight at 4°C. The cleaved Δ51-RGS4 was then isolated by purification over an ANX column (GE Healthcare; Fairfield, CT) in 50 mM HEPES at pH 6.8 and 50 mM NaCl. The flow through, containing the ∼99% Δ51-RGS4 as determined by SDS-PAGE gel, was then collected and concentrated using a YM-10 centrifugal concentrator (Millipore; Billerica, MA). The concentration of Δ51-RGS4 was calculated based on the absorbance at 280 nm utilizing a Take-3 plate (Biotek; Winnoski, VT) in a Synergy 2 plate reader (Biotek; Winnoski, VT).

Human RGS8 expression and purification was performed similar to other RGS8 purifications previously reported [27]. An RGS8 truncated construct analogous to the RGS4(Δ51) construct described above, amino acids 60–198 with a C-terminal 6×His tag in the pET28 vector was expressed in BL21-RIPL E. coli (Stratagene; Santa Clara, CA) cells cultured in Terrific Broth (TB) media. Cultures were induced with 200 µM IPTG at OD_600 nm_ of 2.0 and cultured for 16 h at 18°C. Pellet was lysed, centrifuged, and filtered as described above except in RGS8 Buffer (50 mM HEPES at pH 7.5, 500 mM NaCl, 0.5 mM β-mercaptoethanol). Samples were loaded onto a Ni-NTA column (Qiagen; Hilden, Germany), 3 mL for every 1 L media, and washed with RGS8 Buffer supplemented with 25 mM imidazole. The protein was eluted using 200 mM imidazole and fractions were analyzed by SDS-PAGE gel. Fractions containing RGS8>95% purity were pooled and protein concentration was determined by 280 nm absorbance as accomplished above.

Human RGS17 was expressed and purified as a His-tagged protein in E. coli BL21-DE3 (Stratagene; La Jolla, CA), in the pET28 vector as previously described [5].

Human Gα_i1_ (R178M, A326S) rate-altered variant described in literature, was expressed in BL21-DE3 E. coli, grown in TB media, as a 6×His labeled protein in the pQE80 vector [6]. Expression was induced, at OD_600 nm_ of 1.0, with 100 µM IPTG at 30°C for 16 h. Pellets were lysed, centrifuged, and filtered as described above, but in Gα_i_ Buffer (50 mM HEPES at pH 7.5, 500 mM NaCl, 1 mM β-mercaptoethanol, and 20 µM GDP). The sample was first loaded onto a Ni-NTA column (Qiagen; Hilden, Germany), containing 3 mL of resin for every 1 L of media. The column was first washed with Gα_i_ Buffer supplemented with 25 mM imidazole. Gα_i_ was then eluted from the column with Gα_i_ buffer supplemented with 300 mM imidazole. After analysis by SDS-PAGE gel, fractions that contained Gα_i_ were pooled and dialyzed overnight against Gα_i_ Dialysis Buffer (50 mM HEPES at pH 7.5, 25 mM NaCl, 1 mM β-mercaptoethanol, and 20 µM GDP). The sample was then loaded onto a Q-sepharose column (GE Healthcare; Fairfield, CT) and eluted along a salt gradient from 50 mM NaCl to 1 M NaCl in Gα_i_ Buffer. The resulting peaks were then analyzed by SDS-PAGE for fractions containing >99% Gα_i_. The purified Gα_i_ was then assayed for activity utilizing the [^35^S]GTPγS binding assay [28].

Rat Gα_o_ was expressed in LB media as a fusion protein of glutathione-S-transferase (GST), 6× His, and Gα_o_, in pQLinkGD vector. Expression was induced, at OD_600 nm_ of 0.5, with 100 µM IPTG at 30°C for 16 h. Pellets were lysed, centrifuged, and filtered as described above, but in Gα_o_ Buffer (50 mM HEPES at pH 8, 100 mM NaCl, 10 µM GDP, 1 mM tris(2-carboxethyl)phosphine). The protein was first purified over a nickel charged resin column, 1 mL resin for every 1 L culture. Prior to elution, the column was washed with 20 mM imidazole to clear weak binding contaminants from the sample. The fusion protein was eluted with 250 mM imidazole. Fractions were collected and analyzed by SDS-PAGE gel. Fractions containing the protein of interest were pooled and loaded onto glutathione sepharose column (GE Healthcare; Fairfield, CT), 1.5 mL resin for every 1 L culture. The protein was then eluted with 1 mM free glutathione and analyzed by SDS-PAGE gel. Fractions containing >99% pure protein were pooled for activity determination. The purified Gα_o_ was then assayed for activity utilizing the [^35^S]GTPγS binding assay [28].

### Malachite Green Assay

Stock solutions of each of the 3 components of the developing solution were prepared, which are stable for long-term storage [19]. Malachite solution was prepared by first diluting concentrated sulfuric acid 1∶5 in distilled water. Once the solution cooled to 25°C, malachite solution was prepared by dissolving 0.44 g of malachite green oxalate (Alfa Aesar; Ward Hill, MA) in 360 mL diluted acid and stored at 25°C. Molybdate solution, containing 7.5% ammonium molybdate tetrahydrate (Alfa Aesar; Ward Hill, MA), was prepared in distilled water and stored at 4°C. Tween-20 solution, used to maintain solubility of the phosphate-molybdate-malachite complex, was prepared as 11% (v/v) Tween-20 in distilled water. On the day of use, 2.5 mL molybdate solution and 0.2 mL Tween-20 solution were added to 10 mL of malachite solution and mixed quickly to avoid precipitation of malachite. The final ratio of the Developing Solution (DS) was 50∶12.5∶1 (malachite:molybdate:Tween-20). The peak absorbance was determined by a 2 nm step wavelength scan, using 10 µM Na_3_PO_4_ at pH 7.5 as the negative control.

The malachite green assay involves 5 components, with a 1 min spin at 100×g between each addition. For time-course experiments, the first component was 10 µL Malachite Green Assay Buffer (MGB; 50 mM HEPES at pH 7.5, 100 mM NaCl, 5 mM EDTA, 10 mM MgCl_2_, 0.01% lubrol) into a clear 384-well plate (ThermoFisher Scientific; Waltham, MA) using a MultiDrop dispenser (PerkinElmer; Waltham, MA). The second component dispensed was 10 µL of a 4× stock of RGS4, typically 200 nM to 1.6 µM with the target final concentration of 50 nM to 400 nM, diluted in MGB. After a 30 min incubation, 10 µL of the third component, 4× stock of Gα_i_ diluted in MGB, was dispensed (typically between 4 µM and 80 µM with a desired final concentration 1 µM to 20 µM). After a minimum of 5 min incubation, 10 µL of the fourth component, 4× GTP diluted in MGB, was added at 10 minute intervals from 1–110 minutes. The 0 min time point was excluded due to amount of time required to proceed from GTP addition to quenching with DS. 4× GTP concentrations varied between 0.2 mM and 2.4 mM, with a target final concentration of 50 µM to 600 µM. To terminate the reaction, 10 µL of DS was added to each well using a Microlab Star liquid handling robot (Hamilton Robotics; Reno, NV), to achieve a final ratio 4∶1 (sample:developing solution). Following the spin, the plate was incubated for 25 min before being read at 642 nm for absorbance using an EnVision plate reader (PerkinElmer; Waltham, MA). RGS8 was evaluated similarly to as described for RGS4, with 4× stock concentrations from 20 nM and 800 nM. For each time-course, corresponding GTP only wells were included to account for spontaneous hydrolysis of GTP over time.

Time-course experiments for the RGS17 were conducted using the 5 component mixture, with a 1 min spin at 500×g between each addition. The first component was 10 µL MGB into a clear 384-well plate as previously described. The second component dispensed was 10 µL of a 4× stock of RGS17 ranging between 1 µM to 4 µM with the target final concentration of 500 nM to 1 µM, diluted in MGB. After a 30 min incubation, 10 µL of the third component, a 4× stock of Gα_i1_ diluted in MGB, was dispensed at a concentration of 4 µM into each well with a final target concentration of 1 µM. This was incubated for a minimum of 5 min. Then 10 µL of the fourth component, 4× GTP at 1.2 mM diluted in MGB, was added at 10 min intervals from 1–110 minutes with a final concentration of 300 µM. Reaction was terminated as previously described using 10 µL of DS and absorbance was read at 642 nm.

Malachite green compound activity and Z-factor analysis conducted in 384-well plates utilized optimized parameters as discerned from the time-course experiments. 10 µL of 4× compound or MGB was dispensed into appropriate wells. For single point assay, 160 µM compound was used, and for dose-response assays a series of ½ log dilutions from 100 µM final to 316pM final was used. 10 µL of the optimized 4× RGS4 concentration, 0.8 µM in MGB, was dispensed into all wells. After a spin down at 100×g for 1 min, the assay plate was incubated at 25°C for 30 min. 10 µL of the optimized Gα_i_ concentration, 20 µM in MGB, was dispensed to each well and incubated at 25°C for 5 min. 10 µL of the optimized 4× GTP, 600 µM in MGB, was then added to the samples. After spinning the samples down at 100×g for 1 min, the samples were incubated at 25°C for 75 min. The samples were then stamped with 10 µL of DS and incubated for 25 min before reading absorbance at 642 nm.

1536-well Z-factor analysis and compound library screen were accomplished largely as described for 384-well plates. Initial screen and Z-factor determination was performed in a final concentration of 5.5% dimethylsulfoxide. For 1536-well assays NUNC clear plates were used (ThermoFisher Scientific; Waltham, MA). For the compound library, the diverse set of known biologically active compounds, The Spectrum Library (MicroSource; Gaylordsville, CT), was chosen. Each component was dispensed as 1.8 µL samples into each well using a FlexDrop (PerkinElmer; Waltham, MA). To develop the plates, 1.8 µL of DS were stamped in quadrants using the Microlab Star liquid handling robot. After a 25 min incubation, the plates were analyzed using an EnVision plate reader (PerkinElmer; Waltham, MA) at 642 nm absorbance.

### ALPHA-Screen Counter-Screen of RGS4

Chemical labeling of RGS4 was performed using biotinamidohexanoic acid N-hydroxy succinimide ester (Sigma Aldrich; St Louis, MO). The reaction was carried out at a molar ratio of 3∶1 (label/protein) for 3 h at 4°C in 50 mM HEPES at pH 8 and 100 mM NaCl, similar to as previously described [21]. The reaction was then quenched with 10 µL of 1 M glycine for 10 min at 4°C. The free label was then separated from the desired protein using a YM-10 centrifugal concentrator. Final concentration of RGS4 was determined by 280 nm absorbance of the sample.

To prepare RGS4 for analysis using the ALPHA-Screen assay, RGS4 constructs were first labeled in a 1440 µL sample, diluted in Assay Buffer (AB 20 mM HEPES at pH 8, 100 mM NaCl, 0.1% Lubrol, 1% bovine serum albumin), containing 60 nM RGS4, 14.4 µL streptavidin ALPHA-Screen beads (Perkin-Elmer; Waltham, MA). The sample was then incubated for 30 min, on ice, prior to dilution with AB to 2880 µL. In duplicate, 20 µL of each compound at 120 µM was plated across a white 384-well plate (ThermoFisher Scientific; Waltham, MA). 20 µL RGS4 was then plated into each well and the samples were incubated at 19°C for 30 min prior to the addition of GST-Gα_o_. The final concentrations for RGS4 and compound will be 20 nM and 40 µM respectively.

GST-Gα_o_ was prepared for the assay by creating a 1440 µL labeling reaction, diluted in AB, containing 3 nM GST-Gα_o_, 10 µM GDP, and 14.4 µL anti-GST ALPHA-Screen Beads(Perkin-Elmer; Waltham, MA). The sample was incubated for 30 min on ice. A 40 µL sample was then removed and diluted with 40 µL AB; this is positive control. The remaining 1400 µL is then diluted with 1400 µL AB supplemented with AMF (5 µM AlCl_3_, 5 mM MgCl_2_, 5 mM NaF) to a final volume of 2800 µL. 20 µL of each sample was then dispensed into the each well. The final concentration of the GST-Gα_o_ will be 0.5 nM.

Following the addition of both GST-Gα_o_ and biotinylated Δ51-RGS4, the plates were incubated at 19°C for 1 hr prior to reading using the Synergy 2 plate reader.

### Data Analysis

Data was analyzed using Prism analysis software (Graphpad Software; La Jolla, CA). Initial malachite green assay optimization was accomplished by comparing the fit of both straight line and hyperbolic functions. The fit that mostly closely resembled the data was used to represent the data. IC_50_ values for each compound were determined by fitting the data to a sigmoidal curve, which was used to calculate the IC_50_ value.

## Results

### Optimization of Malachite Green Assay

**Figure 2:**
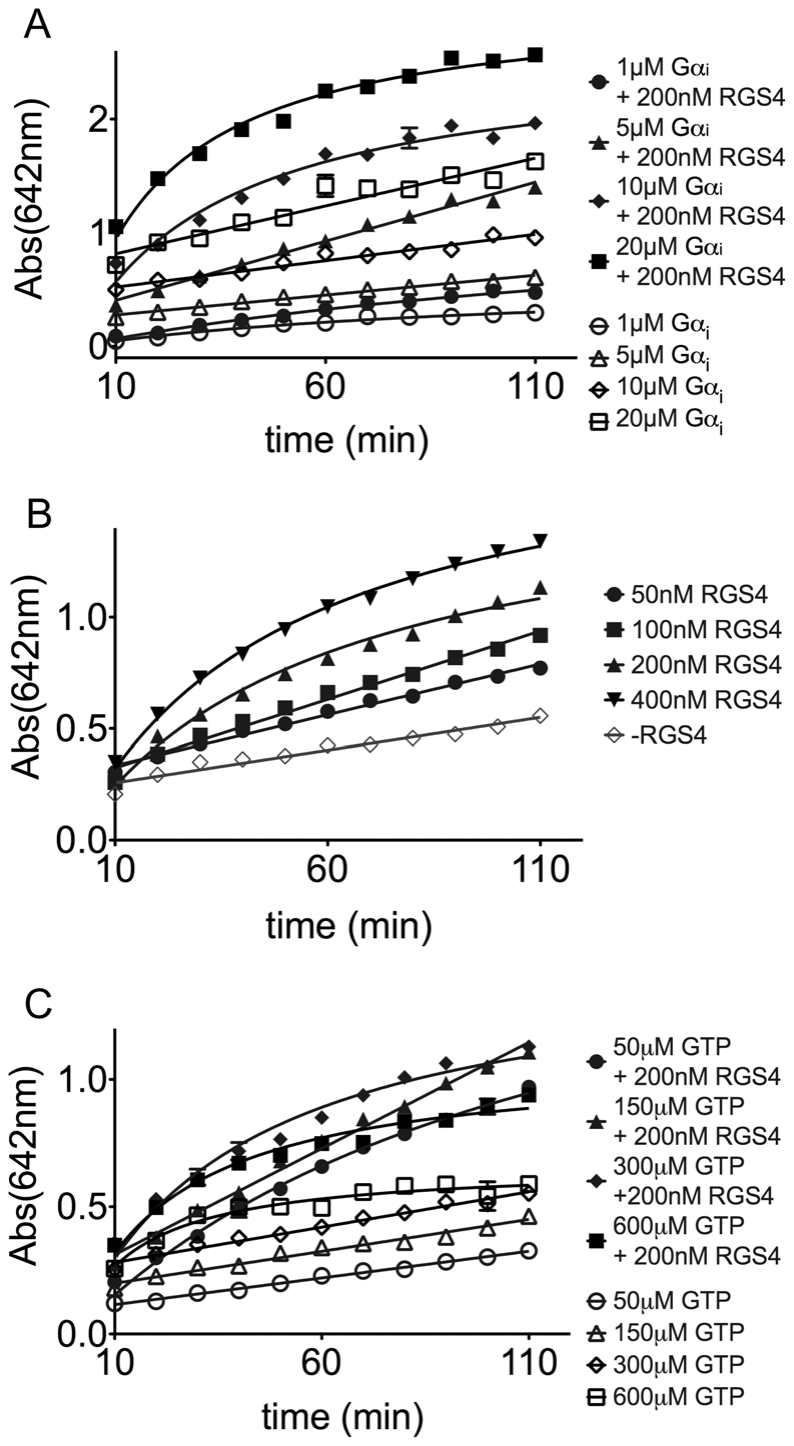
Optimization of Malachite Green Assay for RGS4. (A) Increasing concentrations of Gα_i_, 1 µM to 20 µM final, were compared using final concentrations of 200 nM RGS4 and 300 µM GTP. Absorbance at 642 nm was read every 10 min. Each sample was graphed as with 200 nM RGS4 (closed symbols) or without (open circles). GTP only (300 µM final) control wells were used for background subtraction. (B) Increasing concentrations of RGS4, from 50 to 400 nM final, were compared using Gα_i_ at 5 µM final, and 300 µM GTP. Absorbance at 642 nm was read every 10 min. GTP only (150 µM final) control wells were used for background subtraction. (C) Increasing concentrations of the GTP, from 50 to 600 µM final, were compared using RGS4 (200 nM) and Gα_i_(5 µM), final concentrations. Samples were read at 642 nm absorbance every 10 min.

**Figure 3:**
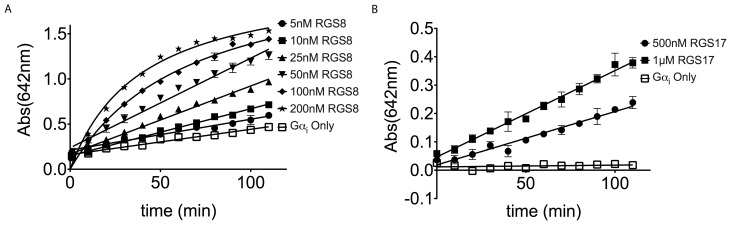
Characterization of Malachite Green Assay with RGS8 and RGS17. (A) Increasing concentrations of RGS8 from 5 nM final to 200 nM final, represented as closed symbols, show signal about equal to 2× the concentration of RGS4, similar to as shown in literature [4]. For comparison, 5 µM final Gα_i_ was included, represented by open symbols. GTP only (150 µM final) control wells were used for background subtraction. (B) Using a Gα_i_ double mutant protein with an accelerated K_off_ for GDP exchange and decrease K_cat_ for GTPase activity we can monitor the effect of RGS17 on the intrinsic GTPase activity of the Gα_i_ subunit. GTP only (300 µM final) control wells were used for background subtraction.

**Figure 4:**
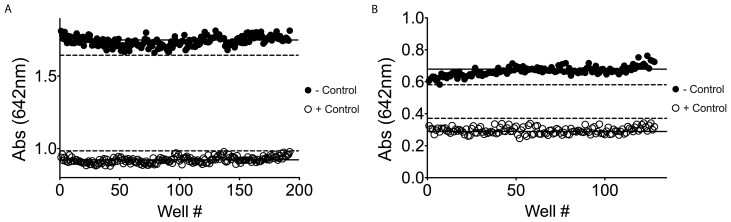
Determination of the Z-factor for 384-well and 1536-well assay. (A) In a 384-well plate, 192 wells were used as a negative control (buffer only), represented by closed circles. An additional 192 wells were used as positive controls and were treated with CCG-50014, a potent RGS4 inhibitor, (10 µM final) represented by open circles [29]. The solid lines represent the mean value for the negative control and the positive control (1.74 and 0.92 respectively). The dashed lines marks the 3 standard deviation cut off for both the positive and negative control (standard deviation of 0.033 and 0.021 respectively). (B) This assay was conducted in 5.5% DMSO to mimic the actual concentration of DMSO in the pilot screen. In a 1536-well plate, 128 wells received buffer, negative control (closed symbols) and the remaining 128 wells received 10 µM final CCG-50014, positive control (open symbols). The solid lines represent the mean value for the negative control and the positive control (0.67 and 0.30 respectively). The dashed lines marks the 3 standard deviation cut off for both the positive and negative control (standard deviation of 0.028 and 0.021 respectively).

The initial focus of these experiments was to determine optimal conditions for the malachite green assay. A wavelength scan of 40 µL of 10 µM Na_3_PO_4_ at pH 7.5 developed for 50 min with 10 µL DS yielded an intense signal peak at 642 nm, with a secondary peak at 436 nm (Figure S1). These peaks coincide closely with the reported literature values of 630 nm and 425 nm [19]. Initial concentrations for each of the components were determined as a ratio of 200 nM RGS4 to 5 µM Gα_i_ based on previously reported ratios [6]. In a time-course evaluation of different concentrations of Gα_i_, higher concentrations of Gα_i_ were excluded due to rapid saturation of the assay, even in the absence of RGS4. Lower concentrations of Gαi proved too slow and provided a small signal window even at 110 min leading to the selection of 5 µM as the optimal final concentration of Gα_i_, as shown in Figure 2A. An added benefit of the higher Gαi concentration is the detection of its intrinsic GTPase activity, marked as open circles in Figure 2A, which allows for an internal control to detect compounds that inhibit Gα_i_ rather than the RGS protein. Having selected 5 µM Gα_i_ as the optimal concentration, we compared a variety RGS4 concentrationswere compared. As shown in Figure 2B, both 200 nM was excluded due to rapid saturation of the assay. Similarly, concentrations of 50 and 100 nM RGS4 proved too slow for our HTS application, generating similar signal windows 1 h slower than 200 nM RGS4 under the same conditions. The final component for optimization, GTP concentration, was evaluated using the selected concentrations of 200 nM RGS4 and 5 µM Gα_i_, as shown in Figure 2C. Higher concentrations of GTP generated increasingly high background, saturating the system early, preventing the development of the high signal window seen previously. For lower concentrations, 50 µM GTP showed substrate depletion as the reaction progressed. Due to similar results between both 150 µM and 300 µM GTP, The lower concentration of 150 µM GTP was selected due to the reduced background signal. From this optimization, the ideal concentrations for RGS4 were determined to be 200 nM RGS4, 5 µM Gα_i_, and 150 µM GTP. For comparison, various RGS8 concentrations were challenged against the optimized Gα_i_ and GTP concentrations of RGS4, Figure 3A, and, as previously reported in literature, RGS8 was about twice as strong a GAP as RGS4, developing a similarly sized signal window with about ½ as much protein [29]. For comparison outside the R4 family, a RZ/A family member: RGS17, was similarly explored. As previously reported in literature, more RGS17 was required to generate a similar signal window, Figure 3B, due to its weak interaction with Gα_i1_ [16]. To confirm the value of this now optimized assay, a comparison of RGS4 with and without 10 µM CCG-50014, a potent inhibitor of RGS4, was used to determine a Z-factor of 0.8, as shown in Figure 4A [29].

### HTS Screen

**Figure 5:**
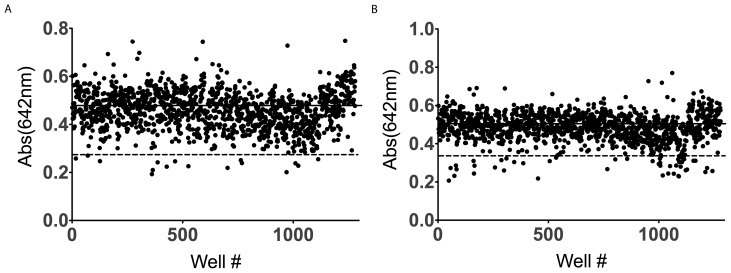
Screen of Spectrum Library. Solid line represents mean negative control. Dashed line represents 3 standard deviations from control and consideration as a hit. (A) In plate one, 16 compounds were identified as initial hits. (B) In plate 2, an additional 43 compounds were identified as initial hits.

Following initial characterization of the assay, the assay was optimized for use in a 1536-well HTS format. Maintaining identical concentrations to the development of the assay in 384-well format, the miniaturized assay yielded a Z-factor of 0.6, Figure 4B. A screen of the Spectrum library was performed in 2 1536-well plates and a final concentration of 40 µM for each compound. Compounds were determined to be hits if they were greater than 3 standard deviations from the mean negative control values. From this initial screen of 2320 compounds, 59 compounds (2.5%) were determined to be hits, Figure 5A and Figure 5B. While this would normally be considered an exceedingly high initial hit rate, the Spectrum Library consists of large set of known biologically active compounds [30].

### Hit Confirmation and Counter-Screen

**Figure 6:**
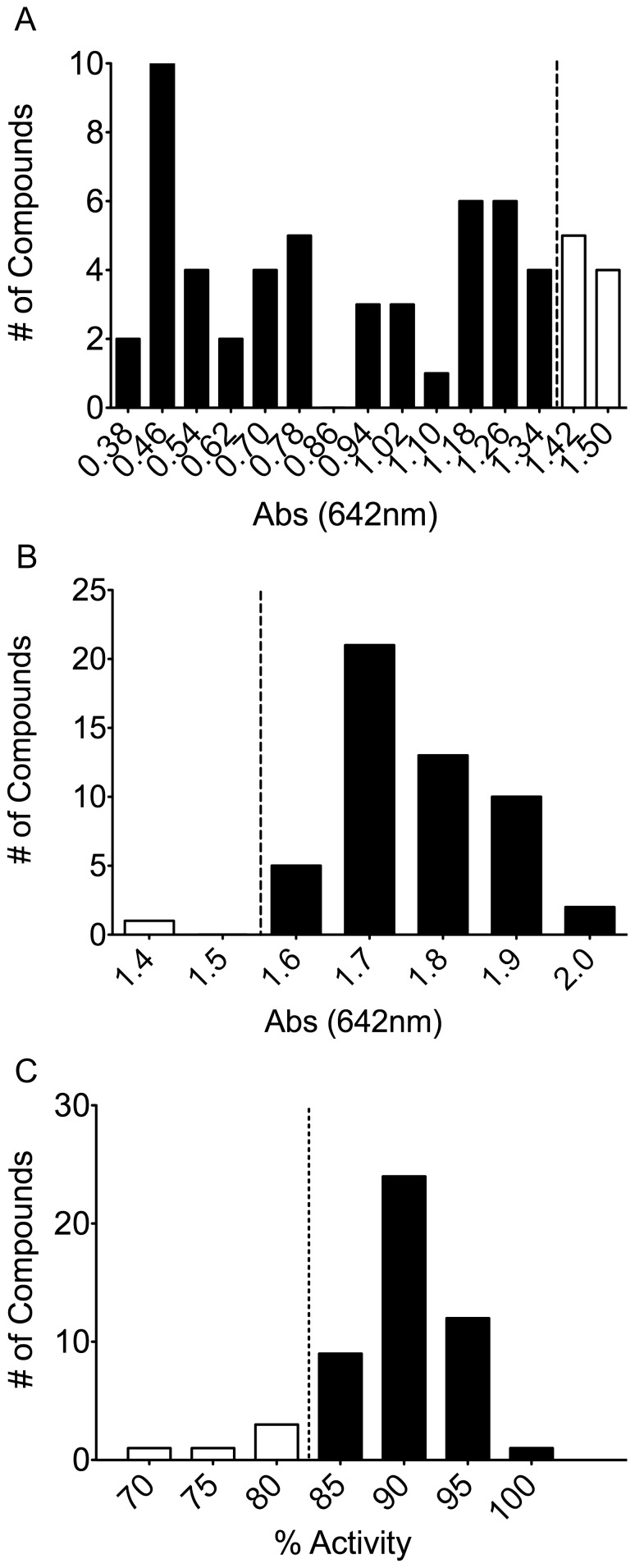
Single Point Hit Confirmation and Control Screens. (A) Single point hit confirmation assay was an analysis of each of the initial hits in a 384-well format (40 µM final for each compound). 7 compounds fell within 3 standard deviations of the negative control and were excluded from further analysis. (B) Phosphate control assay was a comparison of each compound’s (40 µM final) ability to inhibit the assay itself, containing 50 µM phosphate instead of protein. Dashed line represents 3 standard deviations from the negative control. 1 compound fell below 3 standard deviations and was excluded from further analysis. (C) At 40 µM final for each compound, the Gα_i_ control assay evaluated each compound for inhibition of Gα_i_ (5 µM final). The dashed line represents 3 standard deviations below the negative control. 5 compounds fell below 3 standard deviations and were excluded from further analysis. Filled bars represent compounds carried over to following experiments. Open bars represent compounds excluded from further analysis.

**Figure 7:**
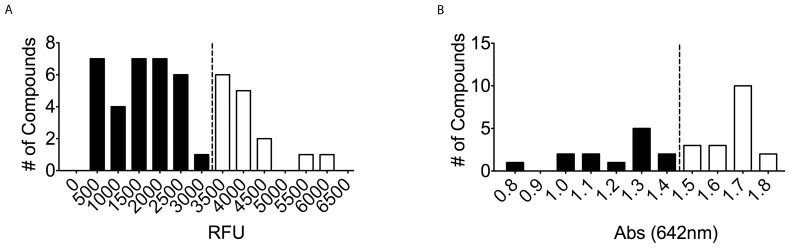
ALPHA-Screen orthogonal assay and RGS4(Δ7) counter screen. (A) At 40 µM final for each compound, this assay was used to confirm each compound as an inhibitor of RGS4 (20 nM final) through another assay. The dashed line represents the cutoff, 3 standard deviations from negative control. 15 compounds fell within 3 standard deviations of the negative control and were excluded from further analysis. (B) This single point assay, at 40 µM compound, was used to confirm activity of each compound against the RGS4(Δ7) mutant (200 nM final). The dashed line represents 25% inhibition, the cutoff for compounds carried to dose-response analysis. 18 compounds failed to inhibit the RGS4(Δ7) mutant of RGS4 and were excluded from further analysis. Filled bars represent compounds carried over to following experiments. Open bars represent compounds excluded from further analysis.

Initial hits were confirmed by single point malachite green assay at 40 µM compound. Of the initial 59 compounds, 7 compounds fell within 3 standard deviations of the negative control, Figure 6A, leaving 52 compounds (2.2%). The assay was followed up with an interference assay designed to test for inhibition of the detection method using 50 µM Na_3_PO_4_ at pH 7.5 to mimic the maximum detectable released P_i_ by the assay. This control would detect compounds that either interrupt the detected complex or reduce the molybdate resulting in peak shift outside of the desired wavelength. 1 compound was found to disrupt the assay, Figure 6B. Compounds that increased the predicted absorbance were carried through, as they would indicate false negatives in the assay. A counter-screen focusing on the intrinsic GTPase activity of the Gα_i_ mutant followed, Figure 6C. Utilizing the known GTPase activity of the Gα_i_ mutant, this assay identified compounds that inhibited the Gα_i_ subunit rather than the RGS protein. This assay, conducted at 40 µM compound, identified 5 compounds that interfered with the assay due to the compound falling 3 standard deviations below the negative control, bringing the total to 45 compounds (1.6%) of the screened library. ALPHA Screen was utilized as an orthogonal assay to confirm each of the remaining compounds as hits, Figure 7A. ALPHA Screen has been successfully used to assay RGS-G-protein interactions in literature [5]. The ALPHA-Screen assay functions by measuring the amount of stable complex formed between the RGS protein and the Gα subunit using the transition state mimic AlF_4_^−^. This orthogonal assay eliminated 15 compounds, leaving 30 compounds or 1.3% of the total compounds screened. Finally, compounds were challenged against the RGS4(Δ7) mutant in the malachite green phosphate detection assay, with the desire of eliminating thiol-modifiers similar to those previously discovered in HTS campaigns against RGS4 [23]. Of the 30 compounds remaining, only 13 compounds also inhibited the RGS4(Δ7) mutant, Figure 7B.

### Characterization of Confirmed Compounds

**Figure 8:**
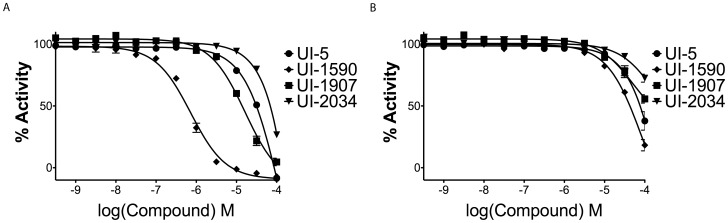
Dose-response anaylsis of UI-5, UI-1590, UI-1907, UI-2034. (A) Inceasing concentrations of compound challenged against RGS4(WT), 200 nM final, in the malachite green assay. (B) The same compounds were compared against the RGS4(Δ7) mutant. All compounds have marked lower potency against the RGS4(Δ7) than the RGS4(WT).

**Figure 9:**
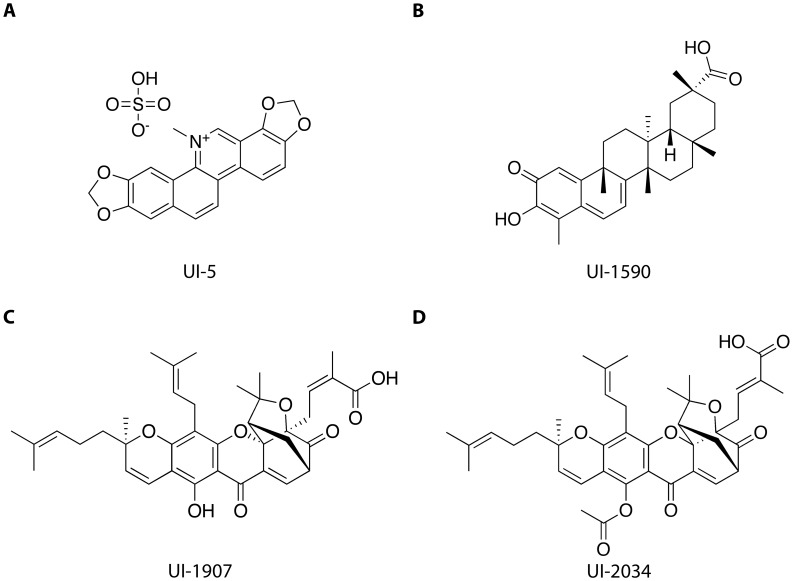
Structure of identified Compounds. (A) UI-5, also known as sanguinarium sulfate. (B) UI-1590 is the pre-therapeutic anti-cancer compound celastrol [40]. (C) UI-1907 is gambogic acid. (D) UI-2034, acetyl-isogambogic acid, is an analogue of UI-1907.

The activity of each of the 13 remaining compounds was assayed by generating concentration-response curves against RGS4 as well as the RGS4(Δ7) mutant. Figure 8A and Figure 8B shows the 4 compounds selected for future analysis. UI-5 (Figure 9A) had an IC_50_ of 126 µM and 454 µM against the RGS4(WT) and RGS4(Δ7) respectively. The most potent compound, UI-1590 (Figure 9B), had an IC_50_ of 724 nM against RGS4(WT) and an IC_50_ of 88 µM against RGS4(Δ7). Finally, two structurally similar compounds, UI-1907 (Figure 9C) and UI-2034 (Figure 9D), had IC_50_ values of 16 µM and ∼269 mM against RGS4(WT), respectively. Against the RGS4(Δ7) mutant, the compounds had IC50 values of 51 µM and 181 µM, respectively. Each of the hit compounds were far less potent against the RGS4(Δ7) mutant than RGS4(WT), similar to what has been reported in literature [4].

## Discussion

RGS proteins are interesting targets due to their role in modulating G-protein signaling. Previous work identifying inhibitors of R4 family RGS proteins have centered on the disruption of the high affinity RGS – Gα interaction observed in the presence of AlF_4_^−^, which mimics the transition state of GTP bound to a Gα subunit [4], [5]. While valid methods for determination of RGS inhibitors, the transition state mimic generated by AlF_4_^−^ generates an RGS-Gα protein:protein interaction with approximately 50-fold higher than basal affinity [31], [32]. The objective of developing this assay was to generate an assay for measuring steady state protein activity that would be economical, fast, easy to use, and adaptable to members of other RGS protein families. The assay developed met each of those criteria.

The initial setup for the assay, for each 1536-well plate, was 1.5 h, which includes incubation steps for the production of free phosphate, allowing the assay to be conducted in highly parallel fashion. Using a colorimetric dye for readout is straightforward and can be accomplished on the simplest of plate readers in absorbance mode. Speed is also essential, and the total read time for each 1536-well plate was only 8 minutes, though this is plate-reader dependent. Perhaps most important is that this assay ameliorates a major concern in high throughput screening – the presence of library compounds that may absorb at a wavelength critical for the assay’s readout. In the case of this malachite green assay, the primary wavelength for the absorption read of the assay is at 642 nm, however, a secondary peak is also present at 436 nm, which provides a second readout to help discriminate compounds that may interfere with the primary readout at 642 nm. The absorbance at 436 nm is lower intensity than that at 642 nm, however it is quite usable as a secondary, confirmatory readout – and one that can be run on the same sample as the primary read (Figure S1 and Figure S2).

**Figure 10:**
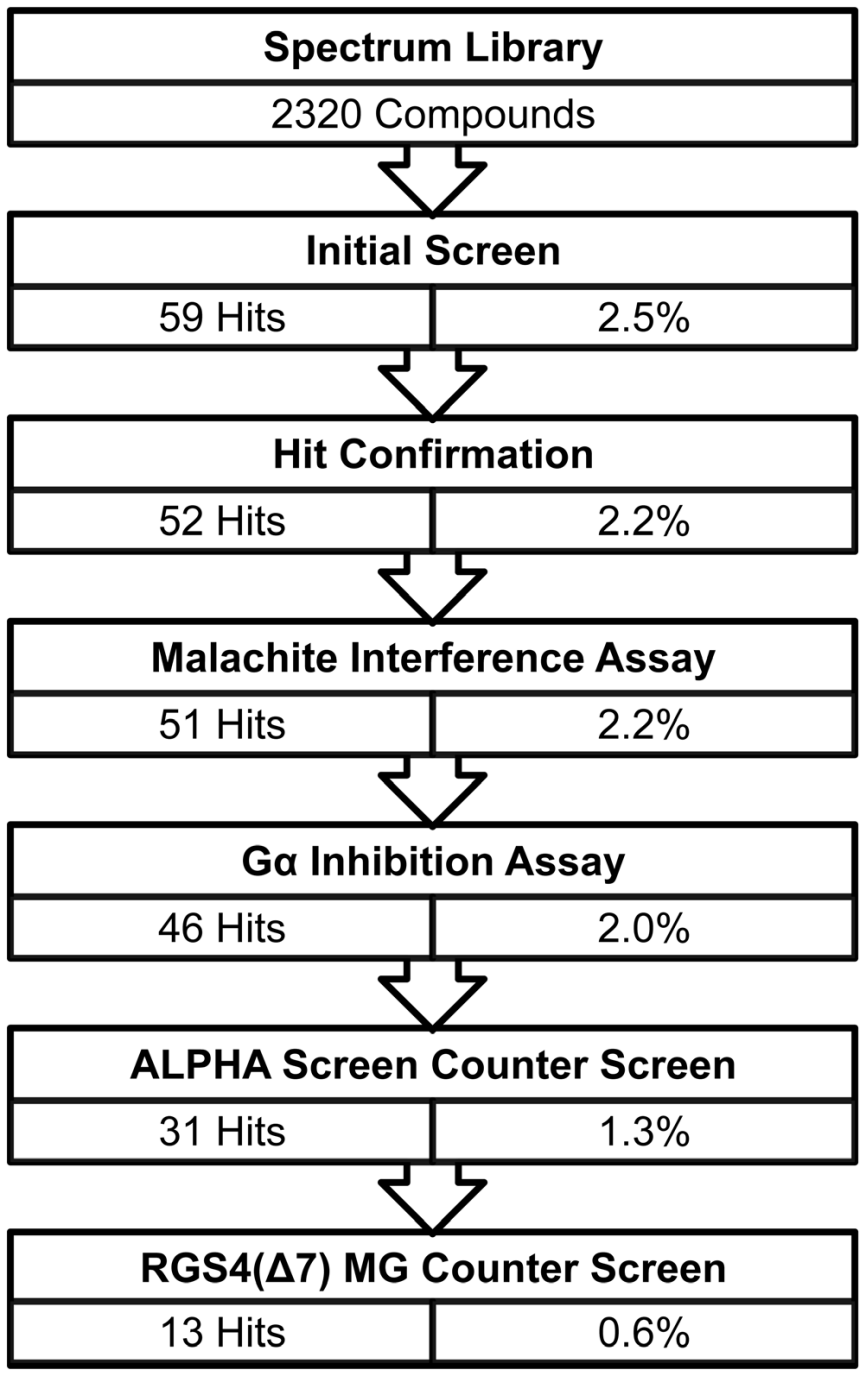
Pilot screen results for Spectrum Library. From the 2320 compound library, 59 compounds (2.5%) were considered hits. 52 of those compounds were confirmed in a single point assay. 6 compounds were found to inhibit either the assay or the Gα_i_ directly, leaving 46 compounds (2.0%). An additional 15 compounds were removed for failing the ALPHA Screen orthogonal assay. And finally, 18 compounds were found to not inhibit RGS4(Δ7) at least 25% in the single point counter screen, leaving 13 compounds (0.6%).

After careful characterization of the constraints of the assay itself, we moved to a small-scale, proof-of-concept screen using a small molecule library of 2320 compounds (MicroSource; Gaylordsville, CT), summarized in Figure 10. The initial results for the 2320 compound library yielded an initial hit rate of 2.5% (59 compounds) that inhibited (by at least 3 standard deviations below the negative control) RGS-mediated GAP activity. RGS-mediated GAP activity is indicated by an increase in free P_i_, generated by hydrolysis of GTP, available to complex with malachite green and increase absorbance at 642 nm. An initial triage included the exclusion of hit compounds that interfered with the assay by directly inhibiting the chemical reactions of the assay readout or inhibiting Gα_i_ itself reduced this hit rate to approximately 2.0%. 7 compounds failed to inhibit RGS4 greater than 3 standard deviations from the negative control in the initial hit confirmation assay using 40 µM compound. 1 compound was found to interfere with the malachite green assay directly, as shown when challenged in an assay containing only 50 µM PO_4_, (greater than 3 standard deviations from the negative control). Finally, an additional 5 compounds were found to inhibit the intrinsic GTPase activity (greater than 3 standard deviations from the negative control) of the Gα_i_ subunit alone. A second, confirmatory screen of the initial hit compounds was performed using an orthogonal assay, ALPHA Screen (Perkin Elmer; Waltham, MA), further reduced this to a hit rate of 1.3% [5]. A Single point ALPHA Screen, using the same concentration as the initial screen, eliminated an additional 15 compounds that failed to inhibit at least 3 standard deviations from the positive control. Of the 31compounds only 13, 0.6% of all compounds screened, were shown to inhibit the RGS4(Δ7) construct (Figure 7B) greater than 25% from the negative control. The RGS4(Δ7) mutant was used as a filter in order to avoid thiol-modifiers similar to compounds already identified previously [22], [23]. These compounds identified in the screen described here were shown weaker inhibitors of the RGS4(Δ7) mutant versus the wild type construct with the exception of two compounds, UI-587 and UI-992.

Each of the compounds demonstrates inhibition of RGS4. Some of the more potent compounds identified, such as UI-587 and UI-662, contain covalent cysteine and free amine chemical functionalities similar to those that have been discovered in other screens against RGS4 [22], [23]. Interestingly, two very similar compounds, UI-1907 and UI-2034, were determined to be weak inhibitors of RGS4 and the RGS4(Δ7). Also identified in this screen is a series of compounds with a quinone functionality, UI-1775, UI-1925, UI-2144, UI-2202, UI-2231, and UI-2249. One of these compounds was the most potent inhibitor of the RGS4(Δ7) mutant, UI-2144. With IC_50_ values from 20–30 µM, UI-1775, UI-2144, UI-2202, and UI-2249 represent some of the most potent compounds reported for the RGS4(Δ7) mutant [4]. Certain compounds, UI-587 and UI-992, inhibited both RGS4 and the RGS4(Δ7) mutant equally. We expected UI-587 to inhibit both equally due to its potential mechanism of action including the modification of free amines. Several of the compounds identified in this screen represent interesting structures, such as UI-5 and UI-1590, and warrant additional investigation, as their mode of action in inhibiting RGS4 is not readily apparent. The most potent compound, UI-1590, is the anticancer drug celastrol which has been studied extensively in both cellular models as well as rodent models, with minor toxicity shown *in vivo* [33], [34].

The development of this assay provides a new method for evaluating RGS proteins and their interactions with G-proteins. Steady-state analysis of RGS activity will allow for more accessible interpretations of compound effects on RGS G-protein interactions. ^32^P liberation assays represent the only well used method for determining the effect of RGS proteins on the rate of GTP hydrolysis. This malachite green assay is capable of almost completely replacing that assay due to its ease of use as well as cost. The mutant used in this assay, Gα_i1_, is capable of being used with a variety of RGS proteins beyond the R4 family, such as the RZ family [35]. Perhaps most importantly, this assay has been shown to be usable with another R4 family member, RGS8, as well as an RZ family member, RGS17. This is promising in that this simple assay should be greatly beneficial for the study of a wide variety of RGS proteins and perhaps other GAPs. Further affording potential for impact in the study of other RGS proteins, the mutations used to generate the mutant G-protein are translatable to a variety of other G-proteins. In Gα_q_, R183C functions very similarly to the mutation R178M in Gα_i1_ [36]. The corresponding mutation in Gα_i2_, R179C, also ablates intrinsic GTPase activity [37]. This highly conserved residue has been shown to be capable of mutation to remove intrinsic GTPase rate but maintain sensitivity to RGS proteins [38]. Similar conserved mutations exist for the rapid exchange of GDP for GTP. One example is the F332A in Gα_t_, which increases the exchange rate by 150 times [39]. Similar conserved residues could be determined in other G-proteins, allowing for expansion of this assay to many more RGS proteins.

In conclusion, we developed a simple, easy to use, and inexpensive assay for the evaluation of the GAP activity of a variety of RGS proteins. This study shows that this colorimetric assay is both robust and readily miniaturized for HTS application. The dual absorbance peak of the assay, 642 nm and 436 nm, allows for an in well counter-screen to include compounds that may have been lost due to absorbance at the primary reading wavelength. The slow but detectable intrinsic GTPase rate of the mutant Gα_i_ allows for a simple counter screen to remove compounds that interfere with the assay by direct inhibition of the Gα_i_ construct. This assay has the potential to expand to encompass a variety of RGS protein families and increase the number of available tools to study this interesting family of proteins.

## Supporting Information

Figure S1 **Wavelength Scan of 10 µM Na_3_PO_4_.** Using 10 µM Na_3_PO_4_ as a control, a wavelength scan of the absorbance of the system was evaluated to determine the optimal wavelength for detection. Two peaks were detected with local maxima at 436 nm and 642 nm.
(TIFF)

Figure S2 **Peak Evaluation.** The two selected peaks, 642 nm and 436 nm, were evaluated using ½ dilutions of Na_3_PO_4_ from 50 µM to 0.4 µM. The peak at 642 nm had a three fold greater response to Na_3_PO_4_ than the peak at 436 nm at equivalent concentrations.
(TIFF)
