# Supplementary figures and images for: A High Throughput Screen for RGS Proteins Using Steady State Monitoring of Free Phosphate Formation
Source: PLoS One. 2013 Apr 23;8(4):e62247. doi: 10.1371/journal.pone.0062247 (PMC3633906; doi:10.1371/journal.pone.0062247)

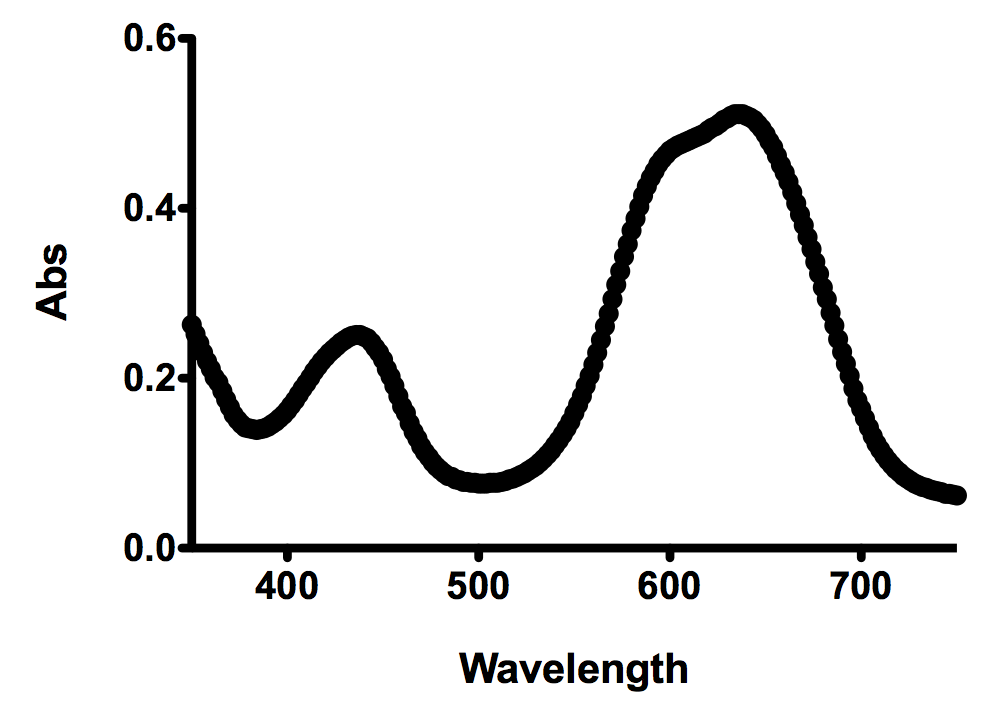

Supplement: Figure S1 — Wavelength Scan of 10 µM Na3PO4. Using 10 µM Na3PO4 as a control, a wavelength scan of the absorbance of the system was evaluated to determine the optimal wavelength for detection. Two peaks were detected with local maxima at 436 nm and 642 nm. (TIFF) [file pone.0062247.s001.tiff]

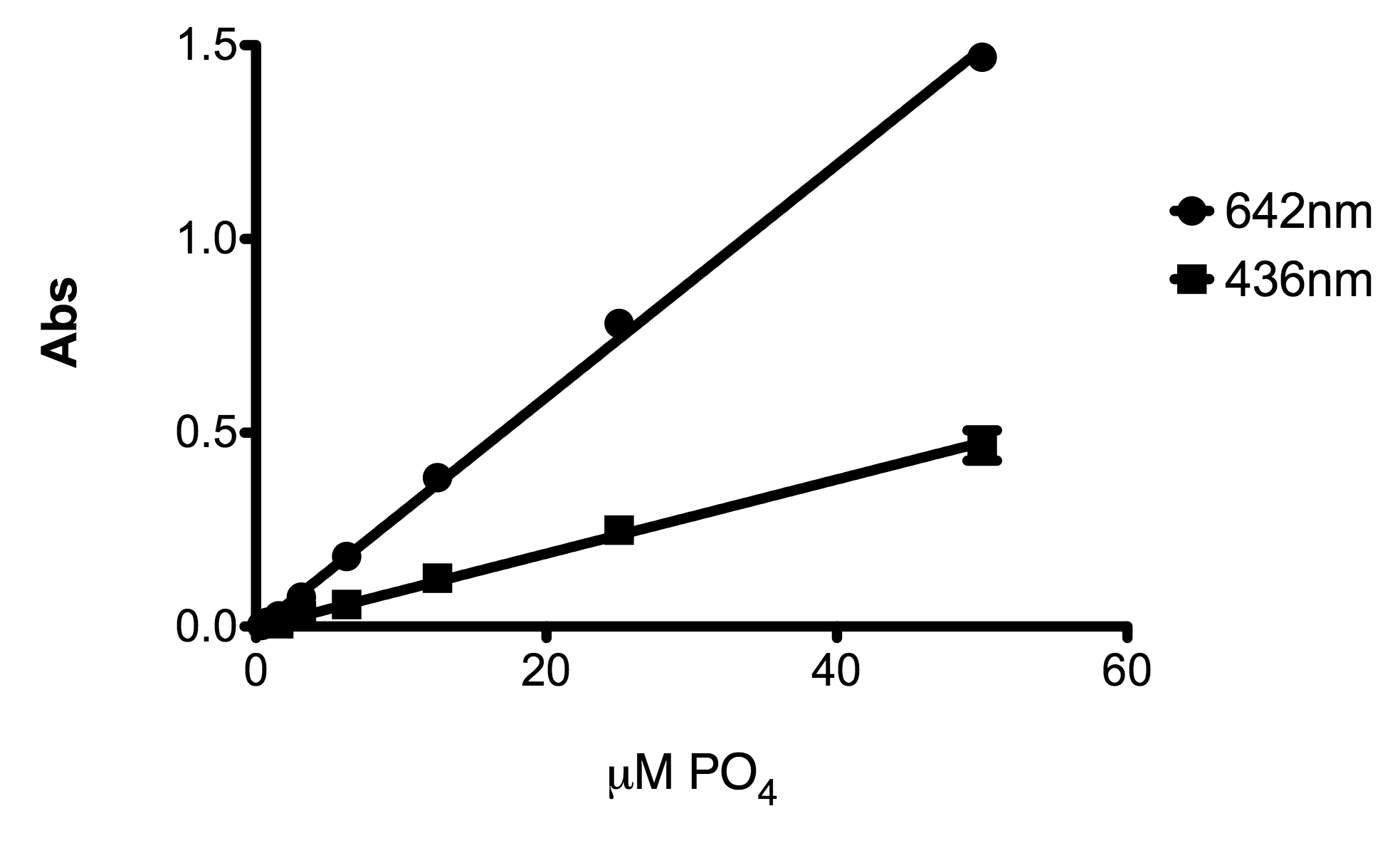

Supplement: Figure S2 — Peak Evaluation. The two selected peaks, 642 nm and 436 nm, were evaluated using ½ dilutions of Na3PO4 from 50 µM to 0.4 µM. The peak at 642 nm had a three fold greater response to Na3PO4 than the peak at 436 nm at equivalent concentrations. (TIFF) [file pone.0062247.s002.tiff]
